# Clinical implication of ivabradine‐incorporated medical therapy for junctional ectopic tachycardia following pediatric cardiac surgery

**DOI:** 10.1002/joa3.13190

**Published:** 2024-11-20

**Authors:** Naoya Kataoka, Teruhiko Imamura

**Affiliations:** ^1^ Second Department of Internal Medicine University of Toyama Toyama Japan

To the Editor,

Junctional ectopic tachycardia (JET) is not amenable to catheter ablation, with amiodarone currently recognized as a recommended therapy for managing JET. However, treating JET presents significant challenges, particularly in patients experiencing hemodynamic instability postsurgery. This study evaluates the feasibility of ivabradine in managing JET following pediatric cardiac surgery, addressing several pertinent concerns.^1^

Accurately diagnosing JET through body surface electrocardiograms alone remains challenging. The authors attempted to rule out atrioventricular nodal reentrant tachycardia by confirming the presence of atrioventricular dissociation or persistent tachycardia following adenosine‐induced atrioventricular nodal block.^1^ However, other arrhythmias, such as infra‐atrial reentrant tachycardia, must also be considered. As these require ventricular overdrive pacing for differential diagnosis, a definitive diagnosis of JET necessitates an electrophysiological study.^2^

In this study, ivabradine was co‐administered with amiodarone in patients with JET and hemodynamic instability.^1^ As intravenous administration of amiodarone can result in hypotension,^3^ ivabradine alone may be particularly suitable for patients with hemodynamic compromise due to its minimal impact on hemodynamics.

The clinical implications of co‐administering a beta‐blocker were not discussed in the study.^1^ Ivabradine is generally indicated for sinus tachycardia and is refractory to the maximum dosage of beta‐blockers. Experimental evidence suggests that ivabradine's efficacy in suppressing the atrioventricular node is reduced under conditions of heightened sympathetic activity.^4^ Therefore, concurrent administration of a beta‐blocker could be practical to maximize the therapeutic impact of ivabradine.

## CONFLICT OF INTEREST STATEMENT

The authors declare no conflicts of interest.

## Data Availability

The manuscript does not include any original data.

## References

[1] Kamali H , Öztürk E , Çiftçi M , Kafali HC , Şahin GT , Haydin S , et al. Use of ivabradine in children with junctional ectopic tachycardia after pediatric cardiac surgery; two‐centre experience. J Arrhythmia. 2024. 10.1002/joa3.13155 PMC11632271 39669919

[2] Tchou P , Nemer D , Saliba W , Varma N , Aziz P , Patel A , et al. Junctional tachycardia: a critical reassessment. JACC Clin Electrophysiol. 2023;9:425–441. 36990601 10.1016/j.jacep.2022.10.040

[3] Cheung AT , Weiss SJ , Savino JS , Levy WJ , Augoustides JG , Harrington A , et al. Acute circulatory actions of intravenous amiodarone loading in cardiac surgical patients. Ann Thorac Surg. 2003;76:535–541. 12902100 10.1016/s0003-4975(03)00509-5

[4] Fontenla A , Tamargo J , Salgado R , Lopez‐Gil M , Mejia E , Matia R , et al. Ivabradine for controlling heart rate in permanent atrial fibrillation: a translational clinical trial. Heart Rhythm. 2023;20:822–830. 37245897 10.1016/j.hrthm.2023.02.012

